# Out of the Blue: A Case of Blue Subungual Discoloration Associated with Prolonged Tetracycline Use

**DOI:** 10.7759/cureus.7810

**Published:** 2020-04-24

**Authors:** Yumna Ahmad, Heidi Boutros, Karim Hanna

**Affiliations:** 1 Internal Medicine, University of South Florida, Tampa, USA; 2 Ophthalmology, University of Central Florida, Orlando, USA; 3 Family Medicine, University of South Florida, Tampa, USA

**Keywords:** minocycline, doxycycline, photosensitivity, hyperpigmentation, subungual, tetracycline, blue

## Abstract

Tetracycline derivatives are antibiotics such as minocycline and doxycycline that have been commonly utilized for inflammatory dermatological conditions such as acne and rosacea. Hyperpigmentation of the skin, nails, thyroid, oral mucosa, teeth, and bones is a known but rare side effect of prolonged tetracycline use. The hyperpigmentation typically takes months to years to develop. There may also be residual changes to the skin after discontinuation of the medication. For this reason, the time tetracyclines are used should be minimized and patients should be monitored for the skin findings. Subungual discoloration carries a broad differential including infectious, inflammatory, metabolic, malignant or systemic diseases. Knowledge of this side effect is crucial in order to avoid unnecessary testing in determining the etiology of the subungual discoloration. We report on a case of a patient who has been on long-term minocycline use for adult acne management. He was initially on minocycline for six years, but due to minocycline-induced hyperpigmentation of his ears and fingernails, he had switched to doxycycline. One year later, the skin hyperpigmentation of the ears regressed; however, the blue subungual hyperpigmentation of his hands progressively become more prominent without any other significant symptoms.

## Introduction

Tetracyclines have been around since the late 1940s and have proven efficacious in treating several conditions [1]. They are noted for their broad-spectrum coverage of bacteria and are widely used because of their bacteriostatic capacity. These medications function by inhibiting bacterial protein synthesis thereby inhibiting bacterial growth. Although tetracyclines are safe to use, there are side effects to be noted. The most commonly reported side effects are gastrointestinal related. They are described to be abdominal pain, nausea, vomiting, and diarrhea. Photosensitivity reactions which typically present as a red rash are also common. Notably, tetracyclines can cause a brown to yellow discoloration in children. Hepatotoxicity is also noted although this is rare. There have also been rare reports of hyperpigmentation of the skin, nails, oral mucosa, teeth, bones, and thyroid gland. Minocycline hyperpigmentation occurs in 2.4%-14.8% of patients and occurs in patients who are on it chronically and on high doses (>100 mg) [2].

The treatment for acne vulgaris typically begins with benzoyl peroxide or topical retinoids. Depending on the severity of the condition, oral antibiotics such as tetracyclines, oral contraceptives, additional topical creams, or oral isotretinoin can be added to the regimen. These therapies target different aspects of the pathogenesis of acne vulgaris [1]. Topical or oral retinoids are used to target follicular hyperproliferation, abnormal desquamation, and increased sebum production. Topical and oral antibiotics treat acne by targeting the proliferation of *Cutibacterium acnes* and mitigating inflammation. The oral antibiotics that are used include tetracycline, doxycycline, minocycline, erythromycin, azithromycin, clindamycin, and trimethoprim-sulfamethoxazole. Doxycycline and minocycline being the most frequently used oral antibiotics for the treatment of acne, due to the increased resistance to other antibiotics.

Hyperpigmentation of the skin, nails, thyroid, oral mucosa, teeth, and bones is a known but rare side effect of prolonged tetracycline use. There are three types of hyperpigmentation reactions. Type I is the most common and is associated with blue-black pigmentations in previous areas of inflammation. Type II is a blue-gray pigmentation of previously normal skin. Type III is a diffuse muddy-brown discoloration of sun-exposed skin [1]. This iatrogenic skin discoloration is cosmetically disfiguring, and early identification is important to prevent further discoloration.

## Case presentation

A 71-year-old gentleman presents to establish care with a new primary care physician after he had lost his prior physician due to a change in his insurance. He has a past medical history of erectile dysfunction, skin cancer, osteoarthritis, and a torn meniscus. He has no acute concerns for his visit besides presenting for an annual check-up. He denies any history of propensity to bleed, tobacco use, or lung diseases that would put him at risk of hypoxia, such as chronic obstructive pulmonary disease, emphysema, or bronchitis. He endorses daily unprotected sun exposure due to yard work and tennis with a prior history of skin cancer. On medication reconciliation, his only prescription medication was doxycycline 100 mg daily. He also takes saw palmetto, fish oil, multivitamin, and methylcellulose.

**Figure 1:**
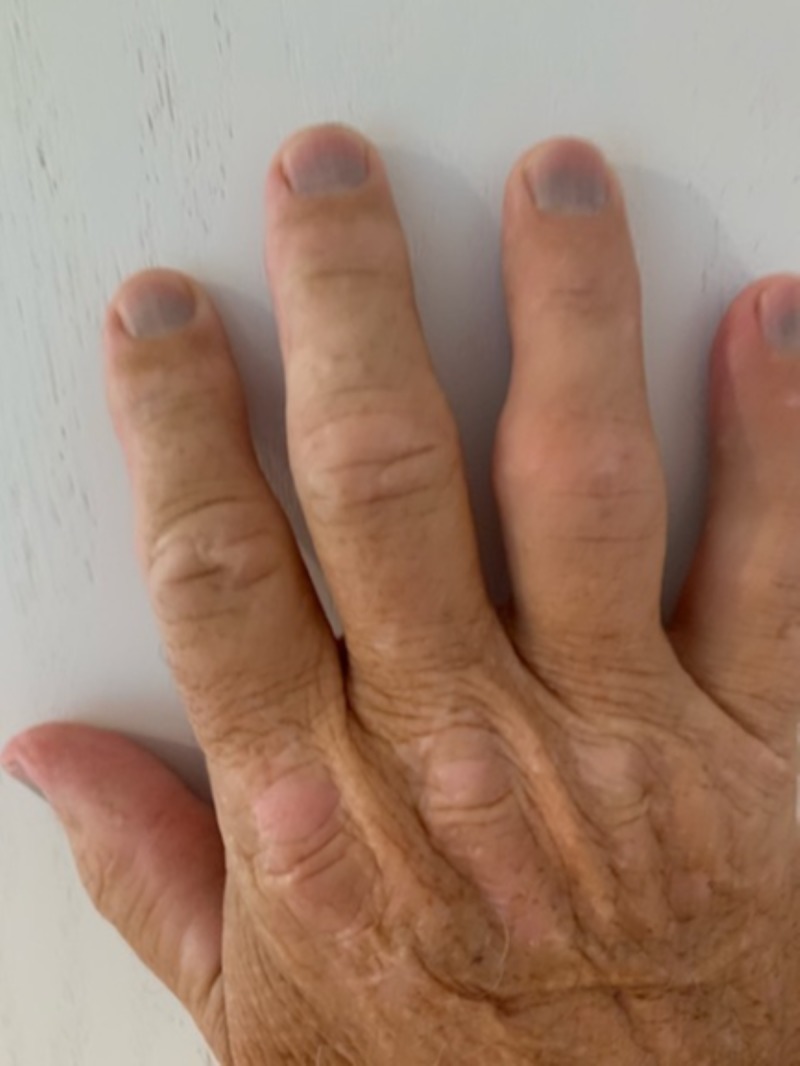
Right Hand Right hand with blue subungual hyperpigmentation

**Figure 2:**
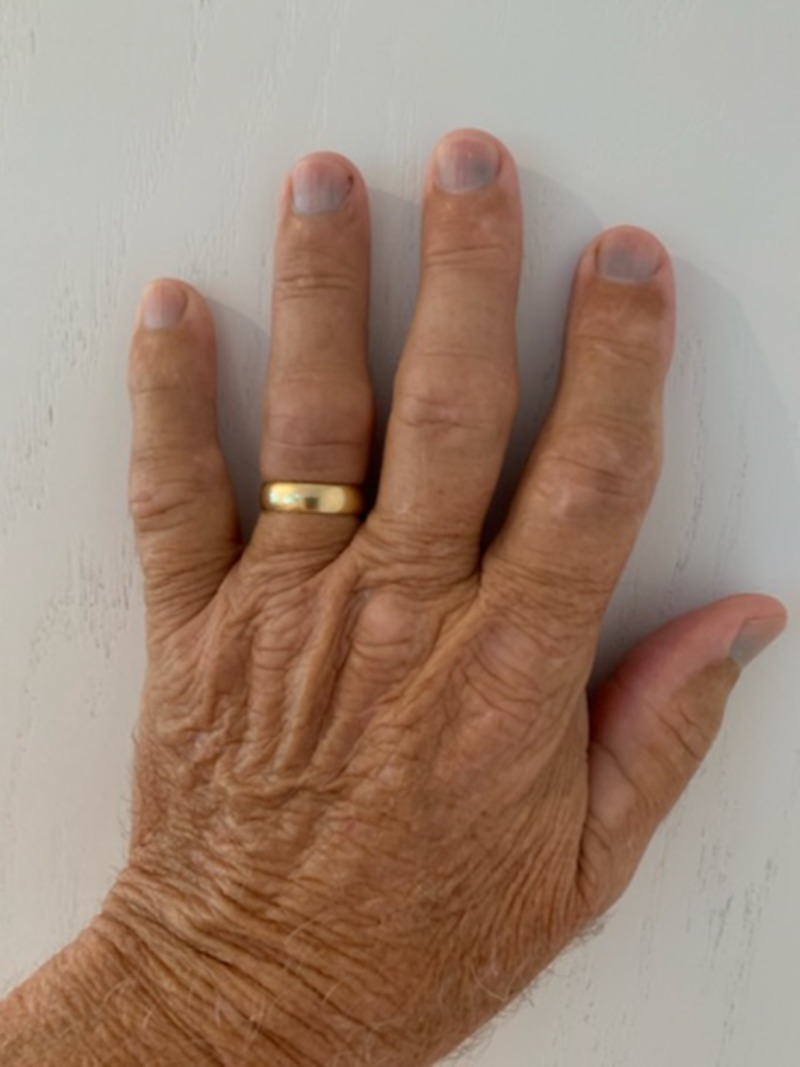
Left Hand Left hand with blue subungual hyperpigmentation

On examination, vitals were stable and pulse oxygen was within normal limits. The exam was benign except for markedly blue-gray discoloration of all the proximal nail beds of both the right and left hands with undisturbed nail formation. This can be seen in Figures 1, 2. This hyperpigmentation spared the toes. He was not cyanotic and capillary refill was normal bilaterally. His fingers were normal temperature to touch, and he denied any change in color in response to cold or stress. His sensation was intact bilaterally. The remainder of his physical examination was within normal limits.

On further questioning, he could not pinpoint when the nail changes began but he mentions that his wife has been noticing it more for the past few years. He denies any pain or discomfort. Although he did have a prior history of skin cancer, it was unlikely that it was a subungual melanoma because of the presence of the pigmentation on all fingers. The patient had no signs of Raynaud's syndrome. On further questioning regarding his use of doxycycline, he had stated that he had been taking minocycline for six years for the treatment of acne vulgaris. However, one year ago he discontinued minocycline and switched to doxycycline due to bilateral ear hyperpigmentation and blue discoloration of his nails. Within that year, the hyperpigmentation on his ears had improved; however, the nail pigmentation did not. The patient denied any recent use of other medications such as amiodarone, antimalarials, heavy metals such as silver, and cytotoxic medications. These medications are known for their blue-hued pigmentation side effect. An in-depth review of systems was otherwise negative.

## Discussion

There have been three types of hyperpigmentation identified by minocycline use. Type I is described as blue-black macules of previously inflamed skin [2]. This is the most common type of skin hyperpigmentation reported with minocycline use. This is a dose-independent reaction and results from local pigment deposits by macrophages. Type II hyperpigmentation is described as previously normal skin exhibiting a blue-gray pigmentation. Type II hyperpigmentation is thought to be a product of pigmented complexes found in dermal macrophages or scattered within dermal collagen. Type III is characterized by a generalized and symmetric muddy-brown pigment of sun-exposed skin areas. This is likely attributable to increased melanin-minocycline complexes at the dermal-epidermal interface [2]. This patient’s distribution is thought to be consistent with type II pigmentation of the nail beds.

Pigmentation of the skin and nails can take months to years to resolve after discontinuation of the drug. Type III is the least likely form of pigmentation to resolve even with discontinuation. It would be advisable for practitioners to warn patients about these risks of discoloration before beginning therapy. In this case, although skin hyperpigmentation was resolved with discontinuation of minocycline, blue subungual hyperpigmentation has persisted and seemed to intensify with doxycycline. Nail bed discoloration induced by doxycycline has been described in a similar distribution as this patient. Akcam et al. reported an 11-year-old boy with nail discoloration caused by doxycycline intake [3]. He had painless brown nail discoloration which disappeared a month after discontinuation of doxycycline. 

The incidence of phototoxic cutaneous reactions with the use of doxycycline is reported to be <5% and has found to be dose-related [4]. It is also reported that patients with lighter skin completion are more susceptible to doxycycline-induced photosensitivity in comparison to patients with darker skin complexion. It is hypothesized that the phototoxic effects are related to the formation of free radicals after being exposed to ultraviolet A radiation in the areas where the tetracyclines are present [4].

## Conclusions

Hyperpigmentation of the skin and nails is a known side effect of minocycline. In this case, the subungual hyperpigmentation did not resolve with discontinuation of minocycline and changing to treatment with doxycycline. Furthermore, the subungual discoloration persisted and intensified while on the new tetracycline. Understanding the possible side effect of subungual hyperpigmentation with the prolonged use of minocycline and doxycycline is crucial to help prevent unnecessary testing and further investigations. Providers should consider this side effect when prescribing these medications for extended periods and consider shortening the duration of delivery. It is also crucial to consider that switching to an alternative tetracycline, such as in this case, may aid in the regression of the skin hyperpigmentation but not nail hyperpigmentation. 
